# Differential Molecular Responses of Rapeseed Cotyledons to Light and Dark Reveal Metabolic Adaptations toward Autotrophy Establishment

**DOI:** 10.3389/fpls.2016.00988

**Published:** 2016-07-14

**Authors:** Dongli He, Rebecca N. Damaris, Jinlei Fu, Jinxing Tu, Tingdong Fu, Chen Xi, Bin Yi, Pingfang Yang

**Affiliations:** ^1^Key Laboratory of Plant Germplasm Enhancement and Specialty Agriculture, Wuhan Botanical Garden, Chinese Academy of SciencesWuhan, China; ^2^University of Chinese Academy of SciencesBeijing, China; ^3^National Key Laboratory of Crop Genetic Improvement, National Center of Rapeseed Improvement in Wuhan, Huazhong Agricultural UniversityWuhan, China; ^4^Wuhan Institute of BiotechnologyWuhan, China; ^5^Sino-African Joint Research Center, Chinese Academy of SciencesWuhan, China

**Keywords:** cotyledons, light, proteome, metabolome, LC-MS/MS, GC-MS, *Brassica napus*

## Abstract

Photosynthesis competent autotrophy is established during the postgerminative stage of plant growth. Among the multiple factors, light plays a decisive role in the switch from heterotrophic to autotrophic growth. Under dark conditions, the rapeseed hypocotyl extends quickly with an apical hook, and the cotyledon is yellow and folded, and maintains high levels of the isocitrate lyase (ICL). By contrast, in the light, the hypocotyl extends slowly, the cotyledon unfolds and turns green, the ICL content changes in parallel with cotyledon greening. To reveal metabolic adaptations during the establishment of postgerminative autotrophy in rapeseed, we conducted comparative proteomic and metabolomic analyses of the cotyledons of seedlings grown under light versus dark conditions. Under both conditions, the increase in proteases, fatty acid β-oxidation and glyoxylate-cycle related proteins was accompanied by rapid degradation of the stored proteins and lipids with an accumulation of the amino acids. While light condition partially retarded these conversions. Light significantly induced the expression of chlorophyll-binding and photorespiration related proteins, resulting in an increase in reducing-sugars. However, the levels of some chlorophyllide conversion, Calvin-cycle and photorespiration related proteins also accumulated in dark grown cotyledons, implying that the transition from heterotrophy to autotrophy is programmed in the seed rather than induced by light. Various anti-stress systems, e.g., redox related proteins, salicylic acid, proline and chaperones, were employed to decrease oxidative stress, which was mainly derived from lipid oxidation or photorespiration, under both conditions. This study provides a comprehensive understanding of the differential molecular responses of rapeseed cotyledons to light and dark conditions, which will facilitate further study on the complex mechanism underlying the transition from heterotrophy to autotrophy.

## Introduction

In rapeseed, large quantities of nutrients are stored in the cotyledon during seed maturation. Proteins, primarily cruciferin (12 s glycoprotein) and napin (1.7 s protein), constitute 20–25% of the dry mass of the mature seed (Ericson et al., 1986). Starch is biosynthesized and deposited transiently, and is eventually converted into oil, which is enveloped in oil bodies containing oleosin embedded in their monolayers. The oil accounts for up to 50% of the dry weight of the seed. Seed germination initiates with water uptake and completes when the radicle protrudes through the seed coat (Bewley, 1997). Proteins are mobilized upon seed germination to provide the embryo with energy and substrates, whereas, the oil reserve is used to fuel postgerminative growth until photoautotroph is established (Penfield et al., 2005; Cernac et al., 2006).

The initial step in oil usage is the breakdown of triacylglycerol (TAG), which is catalyzed by TAG lipase to yield free fatty acids and glycerol (Eastmond, 2006). Free fatty acids are then transferred to the glyoxysome and subsequently catabolized by β-oxidation to generate acetyl-CoA which is eventually converted to sugars by the glyoxylate cycle and gluconeogenesis (Bunkelmann and Trelease, 1996; Graham and Eastmond, 2002). Genetic studies have shown that fatty acid catabolism by β-oxidation is essential for early seedling development, but it does not significantly affect seed germination (Rylott et al., 2006). Glyoxysomes, a special type of peroxisome, are located in storage organs such as fatty seedling tissues. Isocitrate lyase (ICL) and malate synthase (MLS) two key enzymes in the glyoxysome, function exclusively in the glyoxylate cycle. During cotyledon greening, the glyoxysomes are directly transformed into leaf peroxisomes without *de novo* biogenesis of leaf peroxisomes (Titus and Becker, 1985; Nishimura et al., 1986). The increase of photo-respiratory enzymes has been reported to coincide with the marked decrease of glyoxylate cycle enzymes in this process (Titus and Becker, 1985; Nishimura et al., 1986).

The transition from heterotrophy to autotrophy is marked by the rapid transformation of etioplasts to chloroplasts, in which sugar phosphates are synthesized and then catabolized by oxidative metabolism to produce NADPH and ATP for seedling growth. The etioplasts contain prominent lattice-like prolamellar bodies with prothylakoids extending into the plastid lumen (Gunning, 1965). Upon illumination, thylakoids and the photosynthetic apparatus are assembled within a few hours (Lopez-Juez and Pyke, 2005). The genome of the plastid, a semi-autonomic organelle in plant cell, encodes about 80–100 proteins, while 2500–3500 nucleus-encoded proteins are imported to the chloroplast (Abdallah et al., 2000; Peltier et al., 2002). Hence, this light-dependent chloroplast differentiation process requires immediate and coordinated regulation, with multiple organelles being involved in gene transcription, protein translation and localization and subsequent essential metabolic pathways (Albrecht et al., 2006, 2008, 2010; Chen and Thelen, 2010; Rudowska et al., 2012; Albrecht-Borth et al., 2013).

Rapeseed, especially *Brassica napus*, is one of the most widely cultivated oil crops, with worldwide production of over 20 million tons of rapeseed oil annually (ERS, 2015). The genome of *B. napus*, a polyploidy relative of the model plant *Arabidopsis thaliana*, has been completely sequenced and is well annotated (Chalhoub et al., 2014), providing the opportunity to extensively study genetic and biological aspects of this plant and facilitating agricultural breeding. Although a series of studies have reported on the light response of plants during seedling establishment, most of these studies have focused on a single gene or single organelle (Albrecht et al., 2010; Reiland et al., 2011). In the current study, we aimed to comprehensively understand the protein and metabolic modifications that occur in the rapeseed cotyledon during the transition from heterotrophic to autotrophic growth. Using the dimethyl isotope labeling relative quantitation method, we compared the dynamic protein profiles from both the germinated and postgerminative etiolated or greening cotyledons, which was complemented with an analysis of metabolite turnover. Our results showed the differential molecular responses of rapeseed cotyledons to the light and dark, which will facilitate further study of adaptations that occur during the establishment of autotrophic metabolism.

## Materials and Methods

### Plant Material and Seed Germination

Freshly harvested seeds of *B. napus* (Zhongshuang11), with high oil (~ 50%) and low erucic acid content, were washed three times with distilled water. The seeds were then imbibed in distilled water in tissue culture flasks containing two layers of filter paper at 26°C in an incubator in the dark or with 100 mmol⋅m^-2^ s^-1^ white light (16 h light/8 h dark cycle). Seeds germination and postgerminative growth were investigated every day after imbibitions. Cotyledons were collected from rapeseed at 0-6 days after imbibitions for western blot analysis. For the analysis the light/dark response, the seeds were germinated for 1 day under dark conditions and then transferred to the light or kept in the dark for an additional 2 days of growth. Cotyledons from seeds grown for 1 days under dark conditions (D) and two additional days under light (DL) or dark (DD) were collected for following proteome and metabolome analyses.

### Protein Extraction, Digestion and Labeling

Proteins were extracted from cotyledons using the Tris-phenol method as described by Liu et al. (2016). Briefly, the cotyledons (~0.5 g) were ground into a powder in liquid nitrogen using a mortar and pestle, and then dissolved in homogenization buffer (20 mM Tris-Cl [pH 7.5], 250 mM sucrose, 10 mM EGTA, 1% Trion X-100, 1 mM PMSF and 1 mM DTT), followed by centrifugation at 12,000 *g* for 20 min at 4°C. An equal volume of Tris-phenol (pH ≥ 8.0) was added to the supernatant, which was then vortexed thoroughly. After centrifugation at 12000 *g* for 20 min at 4°C, the phenol phase was carefully transferred to another tube, mixed with five volumes of 0.1 M methanolic ammonium acetate (in methanol) and incubated overnight at -80°C. The precipitated pellet was washed once with 0.1 M methanolic ammonium acetate and twice with cold acetone and suspended in lysis buffer (7 M urea, 2 M thiourea, 5% CHAPS, and 2 mM tributylphosphine). The Bradford method (Bradford, 1976) was used to quantify the protein concentrations.

A total of 100 μg protein per sample was pre-fractioned by 12% SDS-PAGE. The visualized gel was cut and subjected to trypsin digestion (protein: enzyme = 50:1 [w/w], overnight at 37°C) as described previously (He et al., 2016). After digestion, the peptides were labeled with isotopic dimethylation reagents using in-solution procedures as described by Boersema et al. (2009). Trypsin-digested peptides from cotyledons of D, DL and DD seeds were labeled with light (2CH_3_), medium (2CD_2_H), and heavy (2^13^CD_3_) dimethyl isotopes, respectively.

### Mass Spectrometry Analysis

The labeled peptides were mixed equally and desalted using ZipTip C18^TM^ (Millipore). After vacuum-dried and re-dissolved in HPLC solution (0.1% formic acid in 2% acetonitrile), the peptides were subjected to spectrometry analyses using a quadrupole-TOF LC-MS/MS mass spectrometer (TripleTOF 5600^+^, AB Sciex) equipped with a nanospray source. Three biological replicates were performed. The peptides were first loaded onto a C18 trap column (5 μm, 5 × 0.3 mm, Agilent Technologies) at a flow rate of 2 μl min^-1^, subsequently, eluted and separated using a C18 analytical column (75 μm × 150 mm, 3 μm particle size, 100 Å pore size, Eksigent). The elution gradient was set at 65 min of 5–23% mobile phase B (Mobile phase A:3% DMSO, 97% H_2_O, 0.1% formic acid; Mobile phase B: 3% DMSO, 97% ACN, 0.1% formic acid), 20 min of 23–52% mobile phase B and 1 min of 52–80% B at a flow rate of 300 nl/min. Survey scans were conducted from 350 to 1500 m/z followed by 40 MS/MS events. The acquired MS data were searched against the *B. napus* database downloaded from http://www.genoscope.cns.fr/brassicanapus/ using ProteinPilot software 4.5 with user-defined search parameters as described by Zhang et al. (2016). The false discovery rate (FDR) thresholds for protein and peptide were specified at 1%, and those with confidence levels higher than 95% were considered. Isotope pattern calculator was implemented to quantify the relative intensities of isotope peaks with Paragon^TM^ Algorithm. The mass spectrometry proteomics data have been deposited in the ProteomeXchange Consortium via the PRIDE partner repository with the dataset identifier PXD003464.

### Western Blot Analysis

About 50 μg proteins per sample were separated by 12% SDS-PAGE and electro-blotted onto PVDF. The blot was probed with Anti-ICL (Sigma, ICL, a marker protein for the glyoxysome), followed by alkaline phosphatase conjugated secondary antibody (Sigma).

### Metabolite Extraction, Derivatization and GC-MS Analysis

Metabolite extraction and derivatization were performed as described previously, with some modifications (Lisec et al., 2015). For each sample, 100 mg of frozen cotyledon powder was added 1.4 ml 85% [w/v] HPLC-grade methanol in MilliQ water and 60 μl 0.2 mg.ml^-1^ ribitol, immediately vortexed and shaken at 1,400 rpm for 10 min at 70°C. The samples were then centrifuged at 11,000 *g* for 10 min. The supernatant was then transferred to a new tube containing pre-cooled 750 μl chloroform and 1.5 ml deionized water and vortexed for 10 s. After centrifugation at 2200 *g* for 15 min, the 150 μl upper layer containing polar metabolites was separated from the subjacent and vacuum-dried. For sample derivatization, 40 μl (20 mg.ml^-1^) methoxyamine hydrochloride in pyridine was added to the dried pellet, followed by incubation at 37°C for 2 h. After addition of 70 μl of *N*-methyl-Ntrimethylsilyltrifluoroacetamid (MSTFA), the mixture was again incubated at 37°C for 30 min with vigorous shaking. After centrifugation at 2,200 *g* for 5 min, the samples were loaded into a 6890 gas chromatograph (GC) coupled to a LECO Pegasus 4D GC × GC-TOF mass spectrometer (MS). GC-MS measurements were performed as described previously, and the obtained raw data were deconvoluted with Chroma TOF^®^ (Doerfler et al., 2013). The peak areas of the same candidate compound from different derived groups were merged. The dry weight frozen cotyledon powder was used to normalize the data.

### Fatty Acid Analysis

About 20 mg of ground cotyledon was added to 1 ml 2.5% vitriol (in methanol) and 30 μl (20 μg) C17:0 standard sample and then esterified at 80°C for 3 h. Then, 1.5 ml 0.9% NaCl and 2 ml hexane containing 0.2% BHT were added and followed by vortexing for 2–3 min. After being centrifuged at 2,000 *g* for 5 min, l ml of the upper layer solution was dried by nitrogen blowing and resolved in 100 μl hexane for GC analysis. A 7820 GC (Agilent Technologies, USA) with DB-23 column (30 m × 0.25 mm i.d., 0.25 μm film) was employed for fatty acid methyl esters (FAME) analysis using helium as carrier gas. The sample was injected in split mode (1:20), the flame ionization detector (FID) temperature was 260°C and the oven temperature program was 150°C for 3 min, followed by an increase of 10°C/min to 240°C and holding this temperature for 5 min. The TAG content was calculated with the following formula, as described by Li et al. (2006): Percent oil by weight = 100 ⋅ ((4 total mol FAME/3) + total g FAME)/g tissue, in which 4 is the relative molecular weight (Mr) difference between TAG and three moles of FAME.

### Bioinformatic Analysis

The statistical significance of the proteome and metabolome data was evaluated by one-way ANOVA with IBM SPSS software (version 20); *p* < 0.05 was considered statistically significant. Protein functions were categorized using MapMan bin codes as described by Usadel et al. (2005). The Kyoto Encyclopedia of Genes and Genomes (KEGG) database was used to annotate protein and metabolic pathways, and the annotation results were mapped on the KEGG pathway database using the online service tool KEGG mapper and pathway tool v19.5. CELLO (version 2.5) was used to predict subcellular localizations. The heatmap was constructed using MeV4.9. GO annotation and enrichment analysis were performed using WEGO^1^ and Agrigo^2^

### Transmission Electron Microscopy (TEM) Analysis

For TEM analysis, the D and DD cotyledons were vacuum-infiltrated and pre-fixed in a solution of 2.5% glutaraldehyde which was adjusted to pH 7.4 with 0.1 M phosphate buffer. Ultra-thin section preparation and TEM observations on a Hitachi H-7650 were performed as described by Han et al. (2013).

## Results and Discussions

### Rapeseed Cotyledon Growth and Isocitrate Lyase Expression

**FIGURE 1:**
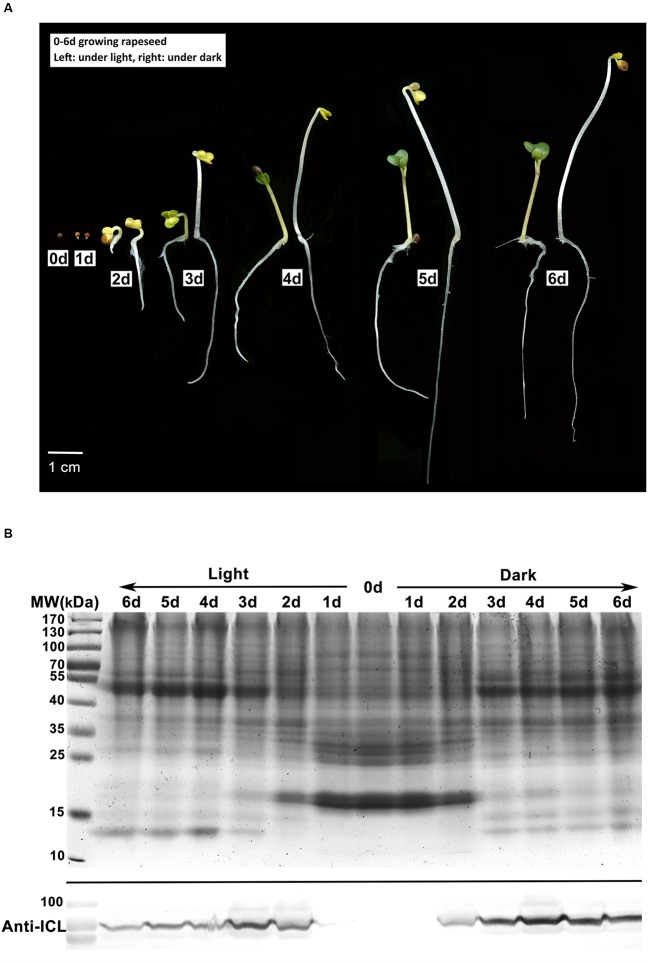
**Characterization of rapeseed germination and postgerminative growth. (A)** Rapeseed grown under light or dark conditions for 0–6 days. Left, under light; right, under dark. **(B)** Western blot analysis of isocitrate lyase (ICL) in cotyledons. Upper, SDS-PAGE gel stained with Coomassie brilliant blue as the loading control, 50 μg protein was loaded for each lane. Lower, proteins were probed with anti-ICL antibody.

To detect the physiological transition from heterotrophic to autotrophic growth, seeds of rapeseed cultivar Zhongshuang11 were germinated under both light and dark conditions. Similar to the finding of Yin et al. (2015), upon imbibition, the appearance of rapeseed seeds was relatively unchanged for the first 6 h. After 12 h of imbibition, emergence of the radicle through the pericarp was observed in about 70% of the seeds. At 24 h imbibition, according to the general definition of seed germination, almost all seeds germinated, and no visible difference was observed in cotyledons between the two conditions. Two days later, yellow cotyledons protruded out of the seed under both conditions. Under dark condition, the hypocotyl extended quickly with an apical hook, and the cotyledons were yellow and folded. By contrast, under light condition, hypocotyl extension was restrained, with reduced hook curvature, and the cotyledons gradually unfolded and turned green in an additional 24 h imbibition (**Figure 1A**).

In young seedlings of oilseed, the stored lipids in the cotyledons are mobilized to fatty acids and then converted to sugars, which provide the primary nutrition for heterotrophic growth until photosynthesis is established (Zimmermann and Neupert, 1980). Fatty acids degradation mainly occurs in glyoxysome which is characterized by two key enzymes of the glyoxylate cycle, i.e., ICL and MLS (Eastmond and Graham, 2001). We investigated the ICL abundance in the cotyledons from 0 to 6 days germinated seeds by western blot analysis. In the cotyledons of dry and 1 day germinated seeds, the ICL signal was negligible. In the seeds grown under dark conditions, ICL levels increased at 2 days after imbibition, accumulated at 3–4 days and remained constant at 5–6 days, whereas under the light conditions, the highest signal appeared at 3 days, followed by a rapid decrease (**Figure 1B**). The results suggests that the glyoxysome activity might be reduced in the cotyledons after 3 days growth under the light, which parallels the cotyledon greening pattern in the light. In contrast, in the dark, the stored lipids might be continually consumed in conjunction with persistent high glyoxysome activity.

**FIGURE 2:**
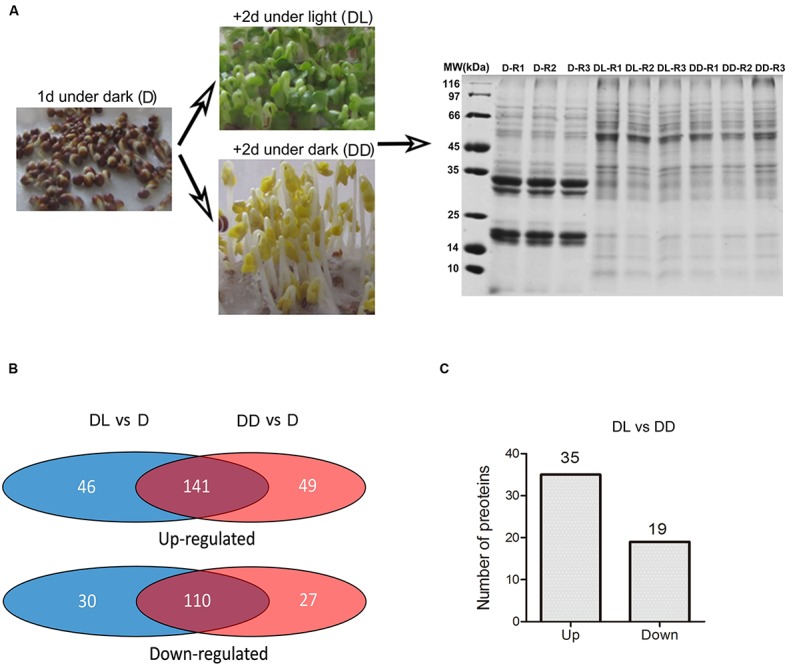
**Framework for the proteome analysis. (A)** Sampling and SDS-PAGE of proteins extracted from the collected samples. Three replicates were prepared for each sample. **(B)** Venn diagram showing the identified differentially expressed proteins. The detailed information was presented in the **Supplementary Table S2**. **(C)** Number of proteins with significant differences in expression (*p* < 0.05, fold change >1.5) between the DL and DD. Detailed information is presented in **Supplementary Table S2**.

Based on the above results, we set the condition of 1 day imbibition in the dark as the starting point to investigate the transition from heterotrophic to autotrophic growth. To investigate the light or dark response of *B. napus* cotyledons, we sampled the globular cotyledons from seeds grown 1 day in dark (D), yellow, folded cotyledons from seeds grown for two additional days in the dark (DD) and green, unfolded cotyledons from seeds grown for two additional days in the light (DL) for following proteomic and metabolomic analyses (**Figure 2A**).

### Proteome Profiling of Germinated and Postgerminative Rapeseed Cotyledons

To explore the light response in the proteome of rapeseed during postgerminative growth, we performed proteomic analyses of D, DD, and DL samples using the dimethyl isotopes labeling method. After protein identification by MS/MS and analysis with ProteinPilot software 4.5 using light dimethyl isotope labeling of D sample as the control, 900, 1021 and 899 proteins were obtained with a FDR of 1% in 3 replicates, respectively. Proteins that were detected in at least two of three replicates were selected for further analysis. In total, 649 proteins were identified. The detailed information is presented in **Supplementary Table S1**. Based on one-way ANOVA (*p* < 0.05) and a fold change in expression (FC) >1.5, 399 proteins were found to be differentially expressed in at least two samples (**Figure 2**), including 141 (21.7%) and 110 (16.94%) proteins commonly up- or down-regulated, respectively, in DL and DD relative to D. Fifty-four proteins were significantly changed in their expressional levels between DL and DD, with 35 up- and 19 down- regulated under light conditions.

**FIGURE 3:**
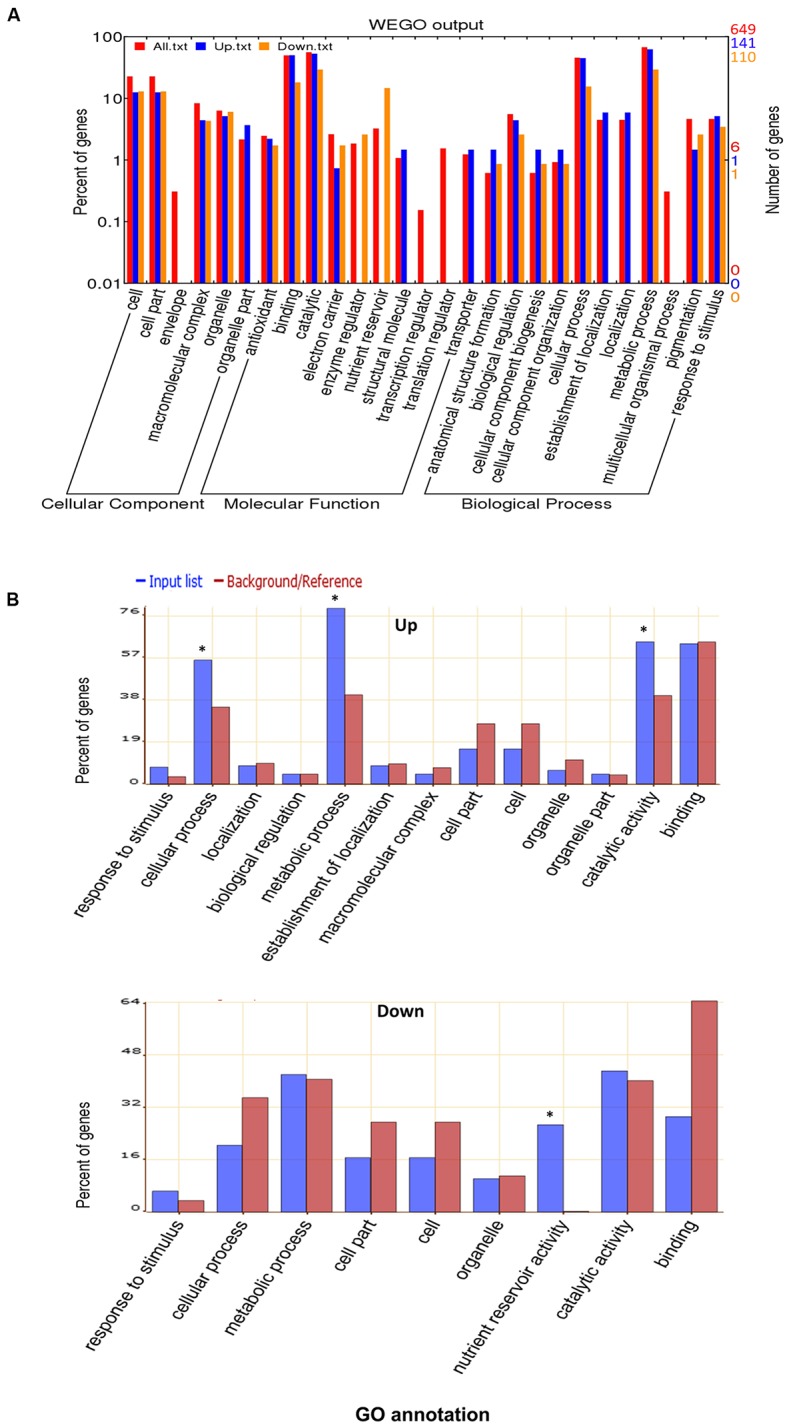
**Gene Ontology (GO) analysis. (A)** GO classification of the total identified proteins. **(B)** GO enrichment for up-regulated or down-regulated proteins in both DL and DD. The online software WEGO and AgriGO were used for GO and GO enrichment analysis, respectively. ^∗^ indicates the significantly different terms.

To elucidate the general light response of rapeseed cotyledons, the total proteins and common up- or down- regulated proteins were classified by Gene Ontology (GO) annotation using WEGO software based on three categories: cellular component, molecular function and biological process (**Figure 3A**). The detailed GO information is listed in the **Supplementary Table S2**. The proteins covered nearly all subcategories of molecular function, the major groups include proteins with binding (303 proteins) or catalytic (355 proteins) related functions, such as proteins catalyzing major/minor CHO metabolism. However, among the up-regulated proteins, none were categorized as nutrient reservoir and enzyme regulation-related proteins. In the biological process category, the major groups were cellular (303 proteins) and metabolic (437 proteins) related processes. Notably, the localization related proteins were missing in the down-regulated group. GO classification revealed the basic makeup of the proteome and indicated that dramatic cellular and metabolic changes occur in cotyledons during early growth.

GO enrichment was further performed to explore the biological functions of differentially expressed proteins using the SEA analysis feature from AgriGO software. As shown in **Figure 3B**, among the down-regulated proteins, nutrient reservoir activity related proteins were remarkably enriched (*p* = 5 × 10^-27^), such as cruciferin, napin and oleosin, which is in accordance with the vast consumption of reserves that occurs at the postgermination stage. For the up-regulated proteins, the cellular process (*p* = 0.00704), metabolic processes (*p* = 2.8 × 10^-6^) and catalytic activity (*p* = 4.4 × 10^-3^) related proteins were enriched, especially photosynthesis (e.g., Calvin cycle, light reaction) and amino acid metabolism-related proteins. These proteins contribute to cellular growth and differentiation during seedling development.

### Metabolome Analysis

**FIGURE 4:**
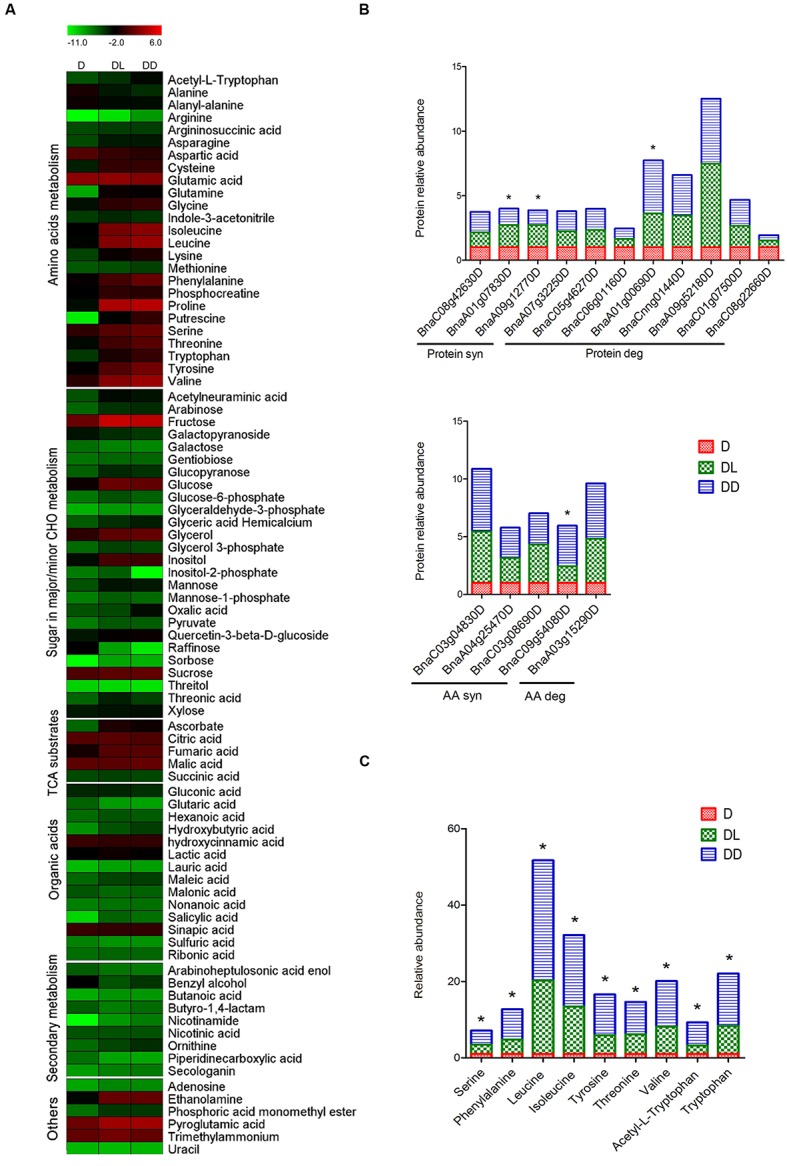
**Metabolites profiling of rapeseed cotyledons. (A)** Heatmap of the dynamic profiling of all identified metabolites. The mean relative quantification data of metabolites were normalized by LOG2. Detailed information is presented in **Supplementary Table S3**. **(B)** Differential expression of protein and amino acid metabolism related proteins. **(C)** Differentially expressed amino acids between DL and DD. ^∗^ indicates the significant difference between DL and DD. Significant expression of both proteins and amino acids were set as *p* < 0.05, fold change >1.5.

To investigate the impact of light on the primary metabolite pool, metabolome analysis was performed using GC-MS. A total of 85 metabolites were detected and quantified, using ribitol as the internal control. The changes in metabolic patterns were highlighted by constructing heatmaps using MEV4.9 software, in which the relative quantification data were normalized by log2 and presented in combination with the KEGG annotations (**Figure 4A**). Statistical analysis revealed that 54 metabolites (62.79%) showed significant (*p* < 0.05, FC > 1.5) changes in abundance between at least two samples. Detailed information is outlined in **Supplementary Table S3**. Most amino acids and reducing sugars were significantly increased, but some non-reducing sugars and organic acids decreased in both of the DL and DD, such as the raffinose and glutaric acid. However, only 14 metabolites were changed between DL and DD, including amino acids.

**FIGURE 5:**
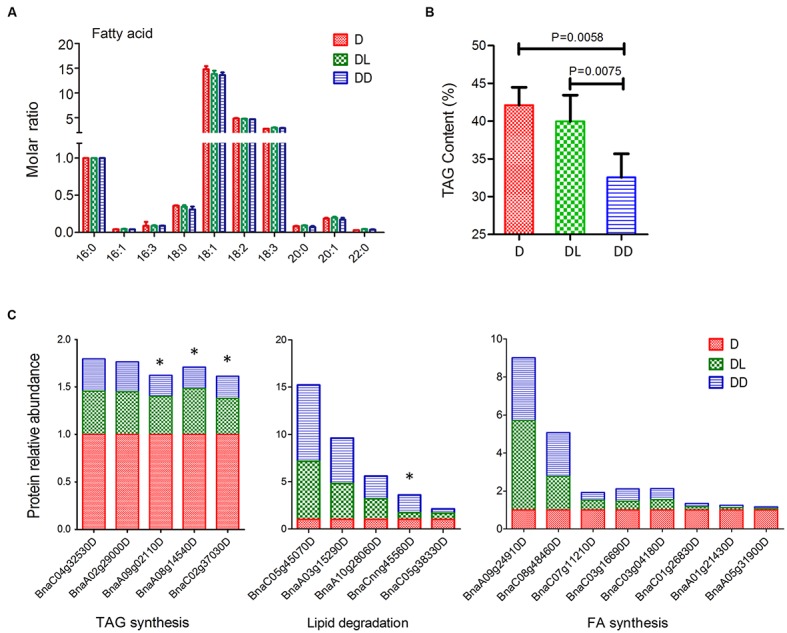
**Fatty acid analysis of rapeseed cotyledons. (A)** Fatty acid composition and their molar ratios in cotyledons. The molar ratio is relative to C16:0 in each sample. **(B)** Total TAG contents of cotyledons. Significance was calculated by *t*-test (*p* < 0.05). **(C)** Expressional changes in lipid metabolism related proteins. ^∗^ indicates significant difference between DL and DD (*p* < 0.05, fold change >1.5).

Mobilization of stored TAG is crucial for postgerminative growth in oil crops. To investigate oil consumption during this process, we determined the fatty acid compositions using GC and quantified their levels with C17:0 as an internal control. All 10 major fatty acids in Zhongshuang 11 were detected. Although there was no significant difference in the molar ratio (relative to C16:0) of fatty acids among the three samples, their TAG contents differed markedly (**Figure 5**). Notably, the TAG content in DL was significant higher than in DD (*p* = 0.0075).

### Light Affects Protein Metabolism in Postgerminative Cotyledons

Cruciferin, napin and the oil body protein oleosin, which account for approximately 50–60%, 20 and 10% of the total seed protein, respectively, are the major storage proteins in seeds, providing the necessary amino acids for *de novo* synthesis of protein during germination and seedling establishment (Hoglund et al., 1992; Huang, 1996). In this study, we found all three major storage proteins rapidly decrease during postgerminative growth, especially under light conditions (**Supplementary Table S1**). The cruciferin and napin proteins decreased more than 10-fold under both conditions. However, the decrease of oleosin was relatively moderate, i.e., about 2–3 fold, especially in DL. Oleosins can associate with triacylglycerides and help stabilize oil bodies in the cell (Hsieh and Huang, 2007). Coincident with the change in TAG content, the relative lower reduction in oleosin content indicates that oil consumption was partially retarded under light conditions.

Other seed development related proteins also decreased, such as the late embryogenesis abundant (LEA) and RmlC-like cupins superfamily proteins. LEAs accumulate significantly in the late stage of seed maturation in response to abscisic acid regulation and function in tolerance to desiccation and other abiotic stresses (Xu et al., 1996). RmlC is a dTDP-sugar isomerase involved in the synthesis of L-rhamnose (Giraud et al., 2000), which might be induced by abiotic stress and participate in pathogen response in plant based on a previous report (Dunwell et al., 2000). The level of some of these proteins decreased relatively slowly, and some (e.g., LEA, BnaA04g25530D) even increased in both DL and DD, suggesting that these proteins might also play roles in stress resistance (e.g., water deficit, salt stress and pathogen infection) during seed germination and subsequent seedling establishment.

Seed storage proteins are mainly degraded through proteolysis. Proteases, especially serine protease and cysteine protease, increased during postgerminative growth. For example, the level of cysteine protease1 (CP1) significantly increased in both DL and DD, and was more abundant in DD. In addition, some ATP-dependent serine-type Clp proteases (e.g., CLPP, CLPC), the most abundant stromal protease during chloroplast development (van Wijk, 2015), increased even under dark conditions. FtsH protease, an ATP-dependent metalloprotease that is directly involved in turnover of the photosystem II (PSII) reaction center D1 protein as part of the repair cycle (Kato et al., 2009), was also abundant in DD. However, the ubiquitin proteasome related proteins (e.g., proteasome beta subunit PBC1 and PBG1) were relatively lower; these proteins might play less important roles in protein degradation at the early stage of seedling establishment. Meanwhile, the levels of some protease inhibitors (e.g., serpin) were also reduced under these conditions.

Along with protein degradation during postgerminative growth, most amino acids increased significantly in the cotyledons, except alanine, aspartate and glutamic acid. Aspartate can be converted to glutamate through the malate-aspartate shuttle, a biochemical system for translocating electrons produced during glycolysis into the mitochondrion for oxidative phosphorylation. Three aspartate aminotransferases (ASP) increased, especially in DD. Meanwhile, two glutamate decarboxylases (GAD) decreased. However, aspartate also decreased significantly, indicating that that it also could be consumed rapidly as a substrate in other metabolic processes. For example, aspartate can be converted into alanine through decarboxylation and further converted into pyruvate. However, alanine significantly decreased during postgerminative growth. Notably, all nine amino acids that were detected differentially expressed between light- and dark- grown cotyledons accumulated at high levels in DD. Consistently, some amino acid biosynthesis related enzymes were significantly up-regulated in DD (**Figures 4B,C**), such as an anthranilate phosphoribosyltransferase (AnPRT, BnaA02g15010D). AnPRT participates in aromatic amino acid biosynthesis pathways specifically for tryptophan biosynthesis. Tryptophan and its derivative acetyl-L-tryptophan, as well as other aromatic amino acids phenylalanine and tyrosine, significantly increased in DD. The increased aromatic amino acids might modify secondary metabolism, such as phenylpropanoids and flavonoids synthesis, and influence seedling development (Ishihara et al., 2006; Dubouzet et al., 2007). Methionine is the first amino acid in protein synthesis and its derivative, *S*-adenosyl methionine (SAM) mainly serves as a methyl donor. Both the methionine synthase and *S*-adenosylmethionine synthetase increased greatly during postgerminative growth, but the methionine content was less variable in the three samples, which likely reflects active translation and metabolic processes occurring in the germinating and postgerminative cotyledons.

*De novo* protein biosynthesis plays a key role in physiological transitions. In the current study, we observed the accumulation of several translational elongation factors and amino acid activation related proteins during postgerminative growth, e.g., RAB GTPase homolog E1B (BnaA01g07830D) and serine-tRNA ligase (BnaC02g39740D). *SCOL1* (*SNOWY COTYLEDON 1*, BnaA09g12770D) encodes the chloroplast elongation factor G. Impairment of SCOL1 affects chloroplast mRNA translation, resulting in the “snowy cotyledon” (Albrecht et al., 2006). The presence of SCOL1 may be one of the prerequisites for the transformation from heterotrophic to autotrophic growth, which helps to explain the observation that SCOL1 levels significantly increased in DL but not in DD.

### Adjustment of Lipid Metabolism and Organic Acids Synthesis Response to Light

**FIGURE 6:**
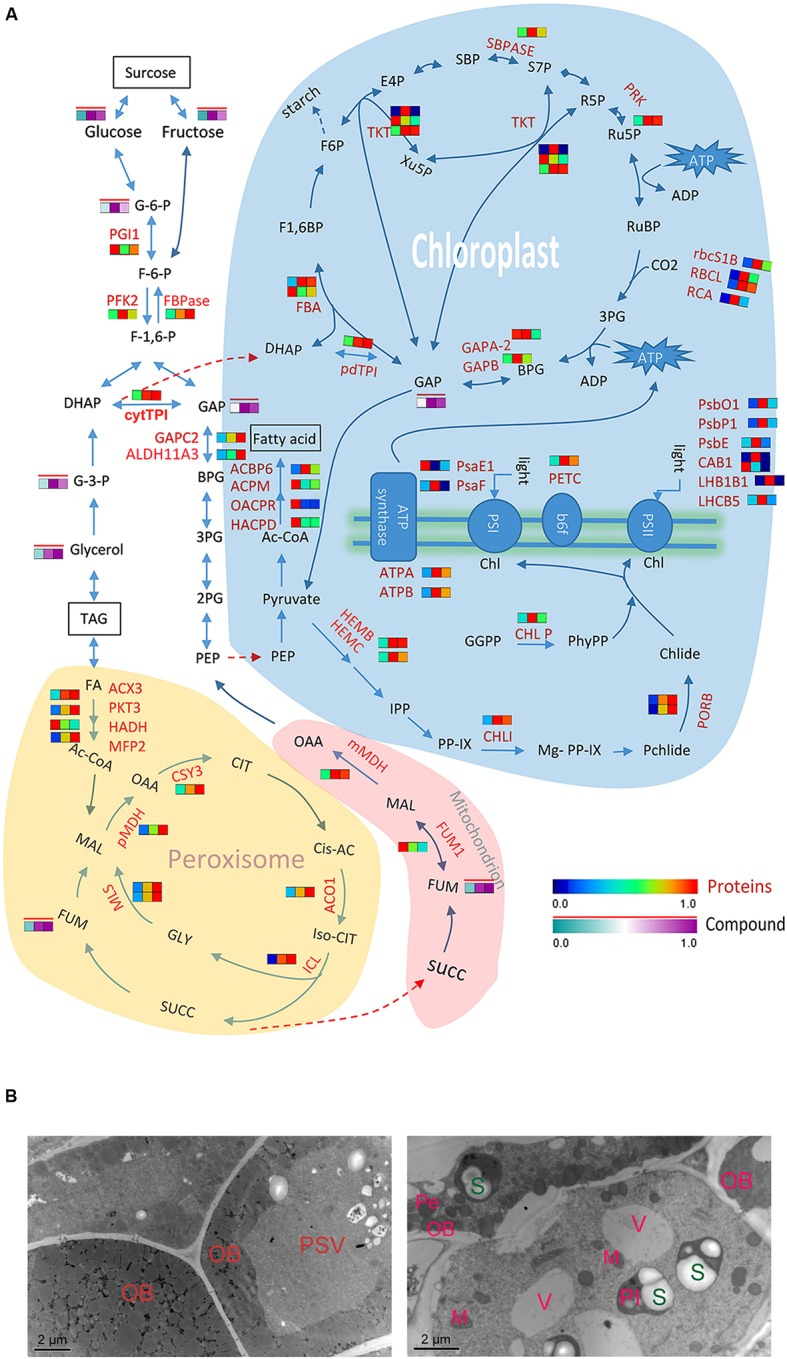
**Differential molecular responses of rapeseed cotyledons to light and dark. (A)** Heatmap showing significantly changed proteins (in a blue-red gradient) or compounds (in a green-purple gradient with a red bar) in cotyledons. Three rectangles in a row from left to right represent proteins D, DL and DD, respectively. Abbreviations: TPI, triosephosphate isomerase; TKT, transketolase; SBPASE, sedoheptulose-bisphos phatase; RCA, rubisco activase; RBCL, ribulose-bisphosphate carboxylase; PSBP-1, photosystem II subunit P-1; PSBO1, PS II oxygen-evolving complex 1; PSBE, photosystem II reaction center protein E; PSAF, photosystem I subunit F; PRK, phosphoribulokinase; PORB, protochlorophyllide oxidoreductase B; PKT3, peroxisomal 3-ketoacyl-CoA thiolase 3; PGI1, phosphoglucose isomerase 1; PFK2, phosphofructokinase 2; PETC, photosynthetic electron transfer C; pdTPI, triosephosphate isomerase; OACPR, 3-oxoacyl-[acyl-carrier-protein] reductase activity; MLs, malate synthetase; MFP2, multifunctional protein 2; MDH, malate dehydrogenase; LHCB5, light harvesting complex of photosystem II 5; LHB1B1, light-harvesting complex II subunit B1; ICL, isocitrate lyase; HEMC, hydroxymethyl-bilane synthase; HADH, 3-hydroxyacyl-CoA dehydrogenase; HACPD, 3-hydro- xyacyl-[acyl-carrier-protein] dehydratase; GAPC2, glyceraldehyde-3-phosphate dehydrogenase C2; GAPB, glyceraldehyde-3-phosphate dehydrogenase B subunit; FUM1, fumarase 1; FBPase, fructose-1,6-bisphosphatase; FBA, fructose-bisphosphate aldolase; CSY3, citrate synthase 3; CLPP5, CLP protease 5; CHLI, magnesium chelatase; CHLP, geranylgeranyl reductase; CAB1, chlorophyll A/B binding protein 1; ATPB, ATP synthase subunit beta; ALDH11A3, aldehyde dehydrogenase 11A3; ACX3, acyl-CoA oxidase 3; ACPM, [acyl-carrier-protein] *S*-malonyltransferase; ACO1, aconitase 1; ACBP6, acyl-CoA-binding protein 6; GGPP, geranylgeranyl pyrophosphate; PhyPP, phytyl-diphosphate; Chl, chlorophyll; IPP, isopentenyl pyrophosphate; PP-IX, Protoporphyrin IX; OAA, oxaloacetate; CIT, citrate; *Cis*-AC, *cis*-aconitate; SUCC, succinate; FUM, fumarate; MAL, malate; GLY, glyoxylate; GAP, glyceraldehyde 3-phosphate; BPG, 1,3-bisphosphoglycerate; G-3-P, glycerol-3-phosphate; 3PG, 3-phospho-glyceric acid; DHAP, dihydroxyacetone phosphate; G-6-P, glucose-6-phosphate. **(B)** TEM images showing the accumulation of starches in cotyledon of at 3 days dark grown seed. Left, 1 day dark grown cotyledon (D); right, 3 days dark grown cotyledon (DD). PSV, protein stocking vesicle; OB, oil body; V, vacuole; Pl, plastid; Pe, Peroxisome; M, mitochondrion; S, starch granule.

In the oil seed crop Zhongshuang11, the TAG accounts for 49% of seed weight. Following seed germination, TAG is broken down into free fatty acids and glycerol. Free fatty acids are then transferred to the glyoxysome and degraded by β-oxidation and the glyoxylate cycle (Graham and Eastmond, 2002). Three major β-oxidation related oxidases, 3-ketoacyl-CoA thiolase 3 (PKT3), multifunctional protein 2 (MFP2) and acyl-CoA oxidase 3 (ACX3), significantly increased in postgerminative cotyledons, and they were more abundant in DD that in DL (**Figure 6A**). However, a 3-hydroxyacyl-CoA dehydrogenase family protein (HADH) also decreased, its function may be substituted by MFP2. Most of the produced acetyl-CoA is involved in the glyoxylate cycle and is ultimately converted to sugars by gluconeogenesis (Bunkelmann and Trelease, 1996). Consistent with the western blot results, ICL increased significantly in DL (8.15-fold) and DD (9.74-fold) relative to D. Five other glyoxylate cycle related enzymes, i.e., citrate synthase 3 (CSY3), two malate synthase, aconitase 1 (ACO1) and peroxisomal NAD-malate dehdrogenase 1 (PMDH1), were significantly increased in DD and DL compared to D, especially in DD. Coincident with the rapid decrease of TAG, the *β*-oxidation and glyoxylate cycle related enzymes increased markedly in the postgerminative cotyledons, especially in the dark.

Succinate produced by the glyoxylate cycle in the glyoxysome enters into mitochondrion and participates in the TCA cycle. Unexpectedly, two TCA related enzymes, malate dehydrogenase (mMDH) and fumarase 1 (FUM1) exhibited the opposite trends during postgermination. FUM1 was significantly decreased in both DD and DL, especially in DD. Correspondingly, the metabolite analysis indicated that the abundance of fumaric acid was remarkably accumulated in DD and DL. Incontrast, other glyoxylate cycle and TCA related metabolites, e.g., the malic acid, citric acid and succinic acid, were not increased during this process. Oxaloacetate from the TCA cycle can be decarboxylated to phosphoenolpyruvic acid (PEP), and transferred into the cytoplast for gluconeogenesis. In the current study, one of the gluconeogenesis specialty enzymes fructose-1,6-bisphosphatase (FBPase) increased in DD and DL. Meanwhile, the levels of the soluble sugars glucose and fructose also increased, especially in DL. These results reflect the active mobilization of the reserves in the cotyledons, which will promote seedling establishment.

### Light Induced Autotrophic Metabolism Establishment in Postgerminative Cotyledons

Seedling establishment requires a transition from heterotrophic to autotrophic growth before the seed reserves are exhausted, and this depends on the recovery of chloroplast function. It was interesting to note that many proteins involved in the chlorophyll biosynthesis and Calvin cycle were significantly increased in cotyledons under both light and dark, even abundant proteins like ribulose-bisphosphate carboxylase (RBCL) and protochlorophyllide oxidoreductase B (PORB). This result implies that these proteins are linked to chloroplast formation in postgerminative growth but note specially induced by light. It also suggests that the role of chloroplasts could be partially performed by etioplast under dark conditions. In green leaves, starch can be transitorily biosynthesized in the chloroplast. A chloroplastic starch biosynthesis related enzyme phosphoglucomutase (PGM) significantly increased under both light and dark conditions in this study. Indeed, our TEM results indicate that starch granules formed in the etioplast, although the thylakoid membranes were difficult to observe (**Figure 6B**). In other plant seedlings, such as rice and soybean (He et al., 2011; Han et al., 2013), a similar phenomenon has also been observed. Light induces great changes in the photosynthetic electron transport chain; several proteins related to photosynthesis system I and II and two ATP synthases significantly increased under light. This result indicates that light directly influences the photosynthetic chain but weakly affects the other systems. This finding may also indicate why 3-day-old etiolated seedlings rapidly turned green and recovered photosynthetic capacities when transferred to the light for only 2 days (data not shown).

Photorespiration is the primary function of the leaf peroxisome, which recycles phosphoglycolate in coordination with enzymes in the chloroplast, peroxisome, mitochondria, and the cytosol (Foyer et al., 2009). In this pathway, the glycolate produced from the oxidation of ribulose in the plastid is further oxidized into glyoxylate by peroxisome-localized glycolate oxidase (GOX), and the hydrogen peroxide generated during this step is detoxified by catalases in the peroxisome. In this study, a GOX increased under both postgerminative conditions. Glyoxylate is then converted into glycine, which is further transferred to the mitochondrion and converted into serine by glycine decarboxylase (GLD), releasing CO_2_ and NH_3_. Indeed, a GLD also increased significantly in both conditions, especially in DL. The released NH_3_ is a valuable resource, which can be efficiently re-fixed and detoxified by glutamine synthetase (GS) in the plastid. Three GSs increased in both conditions, including the GSR1, GS2, and GLN1.3, with GS2 more abundant in DL. These results indicate that light induces the expression of photorespiration related enzymes, but these enzymes might perform other functions besides photorespiration, since they also significantly increased in etiolated cotyledons.

The total lipid level decreased remarkably in seedling cotyledons, which can be ascribed not only to the rapid degradation but also to the low level of *de novo* biosynthesis. Fatty acid biosynthesis related enzymes accumulated during rapeseed seed maturation but degraded rapidly during seedling growth (**Figure 6A**). Therefore, although the substrates, e.g., phosphoenolpyruvate (PEP) from chloroplasts and pyruvate converted from glyceraldehyde 3-phosphate (GAP) in the Calvin cycle, were sufficient for fatty acid biosynthesis, the TAG content were significantly reduced, especially in cotyledons under dark conditions, which had a lower ATP supply for fatty acid biosynthesis.

### Various Systems Employed for Stress Survival

Lipid oxidation and photorespiration result in a burst of reactive oxidative species (ROS), e.g., H_2_O_2_. Although ROS signaling plays positive roles in plastid differentiation after seed germination in conjunction with ABA and NO regulation (Kim et al., 2009; Krasuska et al., 2015), the accumulated ROS is harmful to the cellular structure and metabolism. Several systems are employed in the cell to eliminate oxidative damage, especially for redox related proteins. As the key H_2_O_2_ detoxification system, the glutathione-ascorbate cycle was significantly activated during postgerminative growth. Nine enzymes involved in glutathione-ascorbate cycle increased in both DL and DD. In accordance, ascorbate strongly accumulated, especially in DL. This cycle might appear to operate in the cytoplasm during postgerminative growth, since subcellular predictions indicate that most of the identified proteins were located in the cytoplasm, and few glutathione-ascorbate cycle related enzymes have been found in peroxisomal protein profiles of 3-day-old etiolated cotyledons of *Arabidopsis* (Quan et al., 2013).

Salicylic acid (SA) can induce the accumulation of proline, an effective quencher of ROS biosynthesis and enhancer of plant abiotic stress tolerance (Liang et al., 2013; Khan et al., 2015). SA and proline levels increased rapidly during postgerminative growth. Vacuolar H^+^-translocating pyrophosphatase (VP1) is a proton pump in plants that creates an electrochemical potential gradient across the vacuolar membrane. In rice, *OVP1* overexpression leads to the accumulation of proline, resulting in enhanced cold tolerance (Zhang et al., 2011). Here, we detected significantly higher level of VP1 in DL than in DD, which indicates that more active transport and antioxidant processes occur in the light. At the cellular level, the “protein quality control” network, consisting of molecular chaperones, can reduce protein oxidative modification (Sewelam et al., 2016). The small heat shock proteins (sHSP), which serve as “first responders” to cellular stress, are capable of binding unfolded proteins by an energy-independent process until suitable conditions for protein refolding mediated by ATP-dependent chaperones such as Hsp70 occurs (Sun and MacRae, 2005; Basha et al., 2012). Indeed, we found that sHSPs were more vulnerable compared to other HSPs, for example, four sHSPs decreased significantly in both DL and DD. Mg-ProtoIX, which is sensitive to light and oxidative stress, can interact with HSP81 and other stress related proteins (Kindgren et al., 2011). Five HSP81s were increased in both DL and DD, of which, an HSP81-4 (BnaA03g10790D) was more abundant in DL. The different HSPs might perform functions in stress responses with divergent mechanism. The increase level of these stress related metabolites or proteins should help the plant cope with strong oxidative stresses during postgerminative growth, particularly in DL.

## Conclusion

After germination, the seed proceeds toward seedling establishment, which is marked by the transition from heterotrophy to photosynthesis competent autotrophy. Light plays a decisive role in this switch. To investigate the comprehensive metabolic adaptations that occur during this process, we performed proteomic and metabolomic analyses to demonstrate the differential molecular responses of *B. napus* cotyledons to light and dark. The main results are as follows: (a) Higher levels of ICL accumulated in the cotyledons of 3 days imbibed seed in the light than in the dark, which paralleled cotyledon greening; (b) Light retarded the degradation of reserves in the 3 day imbibed seed cotyledons, implying the successful establishment of autotrophy; (c) Light induced the expression of chlorophyll-binding proteins, cATPase and photorespiration related proteins, but these proteins were also expressed in the dark, suggesting that seedlings grown under dark conditions are also well prepared for the transformation to autotrophy; (d) Various antioxidative systems were employed to confront the stress conditions produced by the dramatic metabolic transition. Although significantly differential responses of rapeseed cotyledons to light and dark were observed, light exerted direct effects on the photo-reaction related equipment prior to autotrophy establishment, and the dark grown seedling maintained the potential toward autotrophy until the reserves were depleted.

## Author and Contributions

DH, RD, CX performed western blot, proteome and TEM analyses; JF, WW, DH performed the metabolome analysis; BY and DH processed the data and wrote the manuscript; JT, TF provided the reagents and materials. PY designed the experiments and revised the manuscript. All authors have given approval to the final version of the manuscript.

## Conflict of Interest Statement

The authors declare that the research was conducted in the absence of any commercial or financial relationships that could be construed as a potential conflict of interest.

## Footnotes

1 http://wego.genomics.org.cn/

2 http://bioinfo.cau.edu.cn/agriGO

## Supplementary material

TABLE S1 **The general information of all identified proteins and their expressional patterns in three samples in this study**.

TABLE S2 **Gene ontology (GO) information of all identified proteins and up-/down-regulated proteins in DL/DD compared with D**.

TABLE S3 **All compounds detected in this study and their expressional changes in three samples**.
